# Emotion Priming in People with Williams Syndrome

**DOI:** 10.3390/brainsci13030467

**Published:** 2023-03-09

**Authors:** Ching-Fen Hsu, Pei Lv

**Affiliations:** 1 Laboratory for Language Pathology and Developmental Neurosciences, Hunan University, Changsha 410082, China; 2 School of Foreign Languages, Hunan University, Changsha 410082, China

**Keywords:** Williams syndrome, emotion priming, emotion categorization, emotion hierarchy, socioemotional cognition

## Abstract

Emotion categories configure the basic semantic knowledge of the human cognitive structure. Previous studies with people with Williams syndrome (WS) investigated their ability to process basic emotions and the dimensions of emotional valences. However, little is known about the categorization of emotions from the subordinate perspective of lexical words in people with WS. In this study, emotion priming was used as the research paradigm. Three types of emotional valence were used as stimuli: positive, neutral, and negative. Each emotional valence was used as a prime matched to a target in one of these same three types of emotional valence. All participants were asked to judge whether the prime and the target were matched in their emotional valence. People with WS (n = 14, 11M/3F, CA = 10.49, and MA = 6.57) showed priming patterns for emotion valences like those of the typically developing controls. When positive primes were presented, accuracy was higher for positive and negative targets than neutral targets. When neutral primes were presented, accuracy was highest for negative targets. When negative primes were presented, accuracy was the lowest for negative targets. All participants showed high priming accuracy for positive emotions; however, they confused neutral with positive targets. A negative priming effect was observed when negative primes preceded negative targets. Considering previous findings that people with WS show developmental delays in the basic emotions of *anger* and *surprise*, this study concludes that people with WS responded least accurately to the classification of emotional valence. The findings regarding the categorization of emotions in people with WS not only advance our understanding of their emotion knowledge and socioemotional cognition but also confirm the superficial enrichment of lexical semantics with weak conceptual change in people with WS. This weakness may result in impaired contextual integration in people with WS.

## 1. Introduction

Emotion is an essential element of cognition, and it is especially related to social cognition, with categorization being an important cognitive ability in humans [1]. Emotion recognition is the most frequently used method to study emotions in people with developmental disorders; it includes both a nonverbal (e.g., face recognition) and a verbal (e.g., language comprehension) domain. Hierarchy in emotion has an evident structure, comprising superordinate, basic, and subordinate levels [2]. The superordinate level is divided into positive and negative valences; the basic level comprises the emotions of joy, love, sadness, anger, and fear; and the subordinate level includes various emotion types such as annoyance, hostility, and resentment, which are categorized as anger. This hierarchy develops from early infancy to adulthood, and emotion develops over time, in turn influencing development [2].

People with Williams syndrome (WS) are populations with genetic deficits on chromosome 7q11.23 and uneven cognitive profiles of relatively good language and poor visuospatial abilities [3]. This syndrome is a rare disease with a traditionally reported epidemiology of 1 in 20,000–25,000 live births [4]. However, at present, the reported epidemiology is 1 in 7500 live births [5]. People with WS are reported to have rich lexical semantics by word generation in the one minute homonymy test [6] and narrations expressed in rich transition words [7]. Given this language proficiency, people with WS have difficulties in social communication and peer relationships. They are characterized as hypersocial, fluent in languages, and advantageous face processors. However, little is known about the categorization of emotions in people with WS. The rationale of this study was to determine the ability of people with WS to categorize emotions based on the processing of affective words. The aim was to confirm a superficial understanding of lexical semantics in people with WS, which may result in impaired contextual integration in this population.

Hsu [8] examined the ability to process emotional valence in people with WS by testing their comprehension of emotional events and words. In the emotion events test, participants listened to disyllabic emotion words denoting events with positive, neutral, or negative valence and made judgments about each target. People with WS were worse at responding to neutral events than the chronologically age-matched (CA) and mentally age-matched (MA) controls, similar to the MA controls when responding to negative events and similar to the CA controls when responding to positive events. Furthermore, the judgment of neutral events as positive events was higher in people with WS than in both control groups, suggesting greater confusion of neutral and positive emotions in people with WS. The MA controls responded to negative events less accurately than the CA controls did. In the emotion words test, people with WS performed similarly to the CA controls, showing the highest and lowest accuracy for positive and neutral words, respectively. The accuracy of responses to negative words in all groups ranged in-between. In general, people with WS were more similar to the MA controls in their responses than the CA controls but had the longest reaction times. Taken together, people with WS had difficulty recognizing neutral emotions, including the comprehension of events and words.

This asymmetry in emotion recognition has also been observed in studies using nonverbal stimuli such as the recognition of facial expressions. In Todd and Porter’s [9] study, using a probe detection task, attention to faces was modulated by facial expressions; the results showed a larger bias among people with WS toward *happy* rather than *angry* faces, whereas the typically developing controls did not show such a bias. This behavioral finding is compatible with neurological results showing higher and lower amygdala activation in response to *happy* and *fearful* faces, respectively, among people with WS [4,10].

Additionally, Hsu and Lv [11] described that the recognition of basic emotions in people with WS differed from that of typically developing controls. In this study, participants listened to narrations related to specific emotions and chose a corresponding emoticon that represented their mental states in target scenarios. Hsu and Lv [11] used the propositions of Ekman [12] for the six basic emotions: *happiness*, *anger*, *fear*, *sadness*, *disgust*, and *surprise*. All participants successfully identified their emotions before the actual experiment began. The results of Hsu and Lv’s [11] research revealed that people with WS recognized the narratives with *anger* and *surprise* emotions similar to the MA controls and the narratives with other emotions similar to the CA controls. Further, when people with WS incorrectly recognized the emotion in the narrative, narratives with *anger* were mainly recognized as being related to *sadness*, and narratives with *surprise* were generally recognized as being related to *happiness*. The difficulty of people with WS in recognizing *anger* in Hsu and Lv’s [11] research coincided with prior findings [9], which showed that people with WS paid less attention to *angry* faces than *happy* faces. Moreover, considering that the recognition ability for *surprise* develops only around 5–6 years old [13] and that the MA controls in Hsu and Lv’s [11] study had an average chronological and mental age of 8.46 years and 5.27 years, respectively, Hsu and Lv [11] posited that people with WS showed delayed performance compared with the MA controls in the experiments.

The findings of Hsu and Lv [11] were concordant with those of Skwerer et al. [14], who reported atypical emotion recognition for both faces and voices in people with WS compared with typically developing controls. Their results also showed that people with WS behaved like people with learning disabilities or intellectual difficulties, showing lower accuracy in recognizing negative emotions in both faces and voices; however, this similarity was not observed when recognizing *happy* emotions. Additionally, emotion recognition was better for faces than for voices across people with WS and healthy controls. People with WS recognized the facial expressions of *sad* emotions better than those of *angry* and *fearful* emotions; they also showed the lowest recognition for *fearful* voices and the highest for *angry* voices. Moreover, the typically developing controls recognized *fearful* and *happy* voices most accurately and showed the worst recognition for *sad* and *angry* voices [14]. In summary, people with WS have difficulty recognizing emotions.

Semantic priming is a transparent task for probing semantic associations in knowledge schemas. It is often considered a useful cognitive paradigm for accessing semantic networks and an automatic process that occurs without conscious control [15]. Word pairs or words in sentences that are semantically related are accessed faster in terms of reaction times than word pairs or words in sentences that are unrelated. Converging evidence from behavioral and electrophysiological studies shows a robust semantic priming effect [16,17] and earlier peaks with a larger semantic priming effect on auditory stimuli than visual stimuli [18]. Hence, in this study, semantic priming was taken as a paradigm to probe the semantic relationship in people with WS.

Previous studies investigated the abilities of people with WS to recognize basic emotions [9,11] and their categorization of emotional valences from the superordinate perspective [8], based on the emotion hierarchy model proposed by Fisher et al. [2]. This study examined how people with WS categorize emotions from the subordinate perspective via priming using auditory input. The researchers hypothesized that the priming effect would be weaker for the same emotional valence in people with WS. By examining the emotion recognition ability of people with WS from the processing of affective words, this study intended to provide more evidence on the deficient socioemotional cognition ability of this population.

## 2. Materials and Methods

### 2.1. Participants

Six groups participated in the emotion priming task. Fourteen participants with WS were recruited and underwent standard measurements of intelligence; the Wechsler Scale Intelligence Test for Children (WSIC-IV) was used for participants younger than 16 years old. In mainland China, no official version of the Wechsler Adult Intelligence Scale has been published or used for participants aged over 16 years. Though the sample of people with WS seems small, this was the maximum number of people that we could recruit during the COVID-19 pandemic period. People with WS were diagnosed in hospital settings as individuals with genetic defects, having an average of 15–22 missing genes at the 7q11.23 region. Participants were matched individually by age and sex.

Fourteen CA and 14 MA healthy participants were recruited. The recruitment of the MA controls was based on the collected intelligence quotients from testing the WSIC-IV, which then transformed the scores into equivalent ages in the norm. This transition was completed individually. These 42 participants were right-handed and native Chinese speakers, and all controls were recruited from the affiliated primary school of Hunan University. No differences emerged upon comparing participants with WS and the CA controls concerning their chronological age or the MA controls concerning their mental age.

Another 20 college students took part in the rating of the stimuli. If emotion priming occurred automatically without conscious involvement, 250 ms inter-stimulus intervals (ISI) would be the more appropriate design; however, if emotion priming was controlled by consciousness, 750 ms ISI should be chosen—compared to 0 ms ISI [200 ms SOA], which was considered as automatic processing, and 550 ms ISI [700 ms SOA], which was considered as controlled processing in Hill et al. [19]). To check whether the emotion priming effect appeared in automatic or controlled processing, another 20 healthy college students in each group (40 participants in total) were recruited for each interval. All 60 of these healthy participants were right-handed, native Chinese speakers, had normal hearing, and were recruited at Hunan University, China. Participants’ background information is presented in Table 1.

### 2.2. Materials and Design

Three types of stimuli were tested: positive, neutral, and negative. All stimuli were selected through the following strict process. To ensure that the stimuli were relevant to the daily lives of all participants and familiar to them, they were selected from the following sources: the first two seasons of the animation *Peppa Pig* (Chinese version), *Baby Bus*, CCTV News, the *People’s Daily* newspaper, the life sitcom *Home with Kids*, the modern Chinese dictionary, entertainment news websites (i.e., the Baidu search engine), and frequent activities that children engaged in when going to the zoo or parks, including Lego-playing, balloon-blowing, and trampoline-jumping activities.

The length of the words ranged from 2 to 3 Chinese characters. Positive words included those that can arouse *happy* or *joyful* emotions, such as 过年 (guo4 nian2; literal meaning, *pass year*; true meaning, *being in the New Year*); negative words included those that induce *sad* or *angry* emotions, such as 家暴 (jia1 bao4; literal meaning, *family violence*; true meaning, *domestic violence*); and neutral words included those that elicit no specific emotions, for instance, 喝水 (he1 shui3; literal meaning, *drink water*; true meaning, *drinking*).

A rating task was conducted after the stimuli were selected. Initially, 648 emotional words were displayed in the rating, including 246 positive, 198 neutral, and 204 negative words. The words were recorded by a woman (CA = 25.3) at 44.1 kHz using the sound-editing software *Praat*. The average length was 2.26 s for positive words (SD = 0.25 s, range = 1.71–2.96), 2.52 s for neutral words (SD = 0.27 s, range = 1.78–3.24), and 2.37 s for negative words (SD = 0.30 s, range = 1.82–3.60). As aforementioned, 20 college students participated in the rating task (mean age = 20.9, SD = 2.3, and 10M/10F). All the words were recorded in neutral prosody without affective interference. All words were presented randomly through *E-prime*.

Among all the rated words, 120 words of each emotional valence were selected as the best stimuli for the emotion priming task (120 of 244 positive words, 120 of 197 neutral words, and 120 of 205 negative words); thus, 360 words were chosen as experimental stimuli. These words were selected based on ranked reaction times for each word, which were ranked separately for each type of emotional valence. Among the 120 experimental stimuli for each emotional valence, 60 words were used as the primes and the other 60 as the targets, with 20 words for each task.

The average length was 1.17 s for the positive primes (SD = 0.18 s, range = 0.84–1.56; mean length of characters = 2.62; SD = 0.49); 1.16 s for the neutral primes (SD = 0.13 s, range = 0.87–1.63; mean length of characters = 2.75; SD = 0.43); and 1.12 s for the negative primes (SD = 0.13 s, range = 0.88–1.54; mean length of characters = 2.13; SD = 0.34). The average length was 1.15 s for the positive targets (SD = 0.17 s, range = 0.84–1.56; mean length of characters = 2.60; SD = 0.49); 1.19 s for the neutral targets (SD = 0.16 s, range = 0.87–1.63; mean length of characters = 2.53; SD = 0.50); and 1.16 s for the negative targets (SD = 0.15 s, range = 0.78–1.50; mean length of characters = 2.40; SD = 0.49).

The results revealed no difference in recorded word length by emotion type among the primes [*F*(2, 118) = 1.93, *p* > 0.05] or among the targets [*F*(2, 118) = 1.017, *p* > 0.05]. A female student (CA = 27.4 years) and a male student (CA = 24.9 years) recorded all words using the sound-editing software *Praat* (16 bit, sampling rate 44.1 kHz).

### 2.3. Procedures

A fixation point appeared on the computer screen for 1000 ms, followed by a prime and a target. After listening to the pair of prime and target words, participants responded as to whether the pair elicited the same emotion. For example, if the first word elicited a positive emotion and the second word elicited the same positive emotion, then a participant should press the key representing the matched emotion. All participants were required to pay attention and listen to the words. No time limit was set for providing responses; however, participants were encouraged to press the corresponding buttons as soon as possible without error. Nine practice sessions were provided to each participant before the experiment began.

The button pressing was counterbalanced, and the stimuli were presented block-wise to ensure the maximum priming effect in participants with WS. Each participant received three blocks to complete the task, with words of different valence as the primes. In tasks 1, 2, and 3, the primes were positive, neutral, and negative words, respectively. Each emotion prime type was paired with three emotion target types. The dependent variables were reaction times and the accuracy of the responses to the three emotion types. The three tasks were run by *E-prime*, and the whole experiment took approximately 20 min to complete. All the words were presented in neutral prosody and without affective interference.

## 3. Results

### 3.1. Strategy for Data Collection and Analyses

Repeated-measures three-way analyses of variance were performed, with the three types of emotion primes (positive, neutral, and negative) and targets (positive, neutral, and negative) as the within-participant factors and the groups (CA, MA, and WS) as the between-participant factors.

Before starting the experiment, two ISIs of 250 ms and 750 ms were tested to examine what would be related to the best priming effect between the primes and targets on emotions. Based on the automatic priming without controlled attention theory [15], the best ISI should be 250 ms; however, this interval was deemed too short for people with WS (based on the priming studies of Hsu [8,20]). To determine the best ISI, studies were conducted on different groups of college students with ISIs of 250 ms and 750 ms. College students’ information is listed in Table 1. The results for ISI 250 ms and ISI 750 ms were not much different; thus, the ISI for this emotion priming study was set at 750 ms to meet the need for revising the research method related to the difficulties of people with WS. The selection of this time interval was also based on a previous study by Hill et al. [19].

The procedures here were the same as those described in Section 2.3. The current results are reported in Section 3.2 for accuracy and Section 3.3 for reaction times.

### 3.2. Data Analysis of Accuracy in the Emotion Priming Study

Accurate matches in the responses to the primes and targets were counted as correct trials and included in the analyses. To make the results clearly displayed, the accuracy for the three groups in responses to the three types of targets given the three types of primes is listed in Table 2.

Regarding the accuracy of the responses when positive primes were presented, the differences by emotion target type were significant [*F*_(2,76)_ = 7.571, *p* = 0.001, *η*² = 0.166]. The accuracy was the highest for negative (0.808, SE = 0.029) and the lowest for neutral emotion targets (0.647, SE = 0.039), and the difference between the two was significant (*p* < 0.001). The accuracy for positive emotions was in-between (0.795, SE = 0.029), and although the responses were less accurate than those for negative emotion targets, the difference between these two was not significant. Moreover, a significant difference was observed between the accuracy of responses to positive and neutral emotion targets (*p* = 0.013).

**Figure 1:**
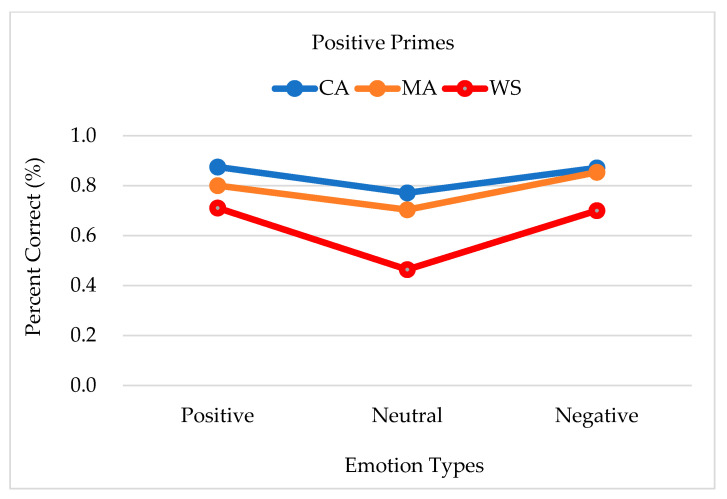
Accuracy of the responses to positive primes by each emotion target type and group. Note. CA, chronological age-matched controls; MA, mental age-matched controls; WS, participants with Williams syndrome.

The group differences in response accuracy when positive primes were presented were significant [*F*_(2,38)_ = 11.704, *p* < 0.001, *η*² = 0.381]. The accuracy was the highest among the CA controls (0.839, SE = 0.033), followed by the MA controls (0.786, SE = 0.034), and the people with WS (0.625, SE = 0.033). The difference between the accuracy of the responses of the MA and CA controls was not significant; however, the difference was significant between the responses of the people with WS and the MA controls (*p* = 0.001) and between the people with WS and the CA controls (*p* < 0.001). No interactions were observed (*F* < 1). Hence, when positive primes were presented, both negative and positive emotions were aroused distinctively. Considering that the responses to neutral emotions were the least accurate, participants seemed to confuse neutral and positive emotions. The accuracy of the responses to positive primes for each emotion target and group is shown in Figure 1.

Regarding the accuracy of the responses when neutral primes were presented, the differences by emotion target type were significant [*F*_(2,70)_ = 10.901, *p* < 0.001, *η*² = 0.237]. Accuracy was the highest for negative emotion targets (0.801, SE = 0.029) and the lowest for positive emotion targets (0.556, SE = 0.043). The accuracy of responses for neutral emotion targets was in-between (0.685, SE = 0.042), and the difference between the accuracy of the responses to negative and neutral emotion targets was significant (*p* = 0.035), as well as between negative and positive emotion targets (*p* < 0.001). As the accuracy of responses to positive and neutral emotion targets did not reach significance, this finding suggests that the positive emotion targets were more similar to neutral emotions for participants, while neutral emotions were processed distinctly from negative emotions.

**Figure 2:**
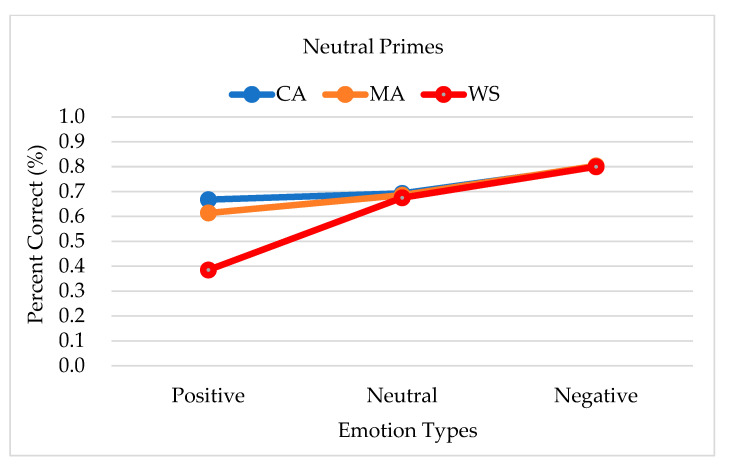
Accuracy of the responses to neutral primes by each emotion target type and group. Note. CA, chronological age-matched controls; MA, mental age-matched controls; WS, participants with Williams syndrome.

Regarding group differences in response accuracy when neutral primes were presented, the accuracy was lowest among the people with WS for all emotion targets (0.620, SE = 0.046), followed by the MA controls (0.701, SE = 0.039), and the CA controls (0.720, SE = 0.039); however, no significant group differences or significant interactions were observed. The accuracy of the responses to the neutral primes by each emotion target and group is shown in Figure 2.

Regarding the accuracy of responses when negative primes were presented, the differences by emotion target type were significant [*F*_(2,76)_ = 12.350, *p* < 0.001, *η*² = 0.245]. Accuracy was the lowest for negative emotion targets (0.472, SE = 0.044), and it was higher and equal for both the neutral (0.690, SE = 0.036) and positive emotion targets (0.690, SE = 0.037).

**Figure 3:**
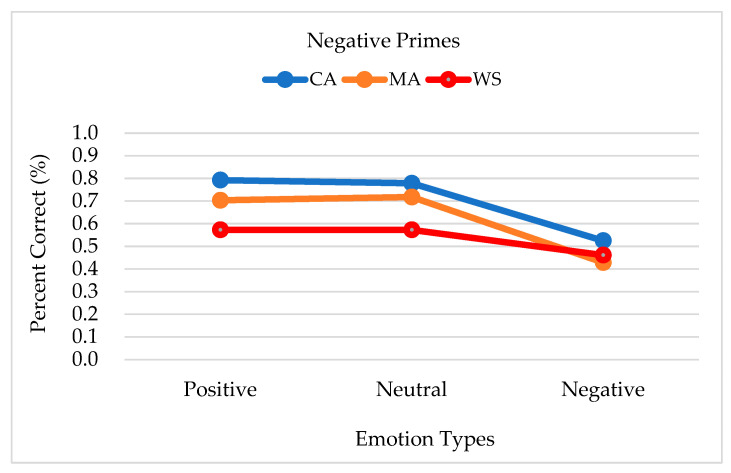
Accuracy of the responses to negative primes by each emotion target type and group. Note. CA, chronological age-matched controls; MA, mental age-matched controls; WS, participants with Williams syndrome.

The group differences in response accuracy when negative primes were presented were marginally significant [*F*_(2,38)_ = 3.208, *p* = 0.052, *η*² = 0.144]. Accuracy was the highest among the CA controls (0.699, SE = 0.045), followed by the MA controls (0.617, SE = 0.045), and the people with WS (0.536, SE = 0.046). No significant interactions were observed. The accuracy of the responses to the negative primes by each emotion target type and group is shown in Figure 3.

### 3.3. Data Analyses of Reaction Times in Emotion Priming

The group differences in reaction times when positive primes were presented were significant [*F*_(2,38)_ = 6.267, *p* = 0.004, *η²* = 0.248]. Reaction times were the longest among the MA controls (5070, SE = 460), followed by the people with WS (3408, SE = 448) and the CA controls (2893, SE = 443). The main effect of emotion target type was not significant (*F* < 1). No significant interactions were observed by group or emotion target type.

The group differences in reaction times when neutral primes were presented were significant [*F*_(2,35)_ = 5.614, *p* = 0.008, *η²* = 0.243]. Reaction times were the longest among the MA controls (5037, SE = 407), followed by the CA controls (3186, SE = 407), and the people with WS (3569, SE = 482). No significant differences were observed between the latter two groups. No significant interactions were observed by group or emotion target type. The differences by emotion target type did not reach significance.

The differences in reaction times when negative primes were presented were non-significant by both group and emotion target type. No significant interactions were observed by group or emotion target type. To make the results clearly displayed, Table 3 is shown for the three groups’ responses to the three types of targets given three types of primes.

**Figure 4:**
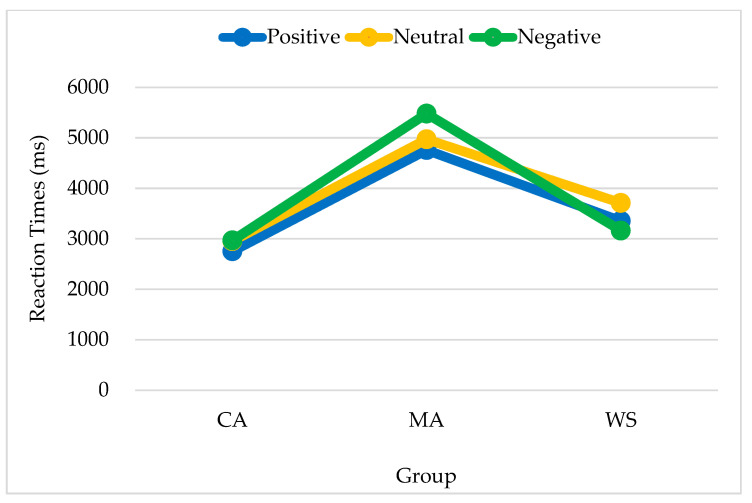
Reaction times of the responses to positive primes by emotion target type and group. Note. CA, chronological age-matched controls; MA, mental age-matched controls; WS, participants with Williams syndrome.

**Figure 5:**
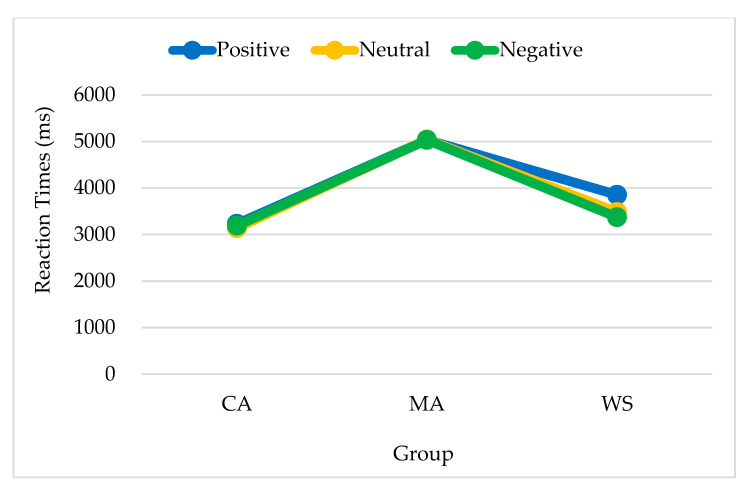
Reaction times of the responses to neutral primes by emotion target type and group. Note. CA, chronological age-matched controls; MA, mental age-matched controls; WS, participants with Williams syndrome.

**Figure 6:**
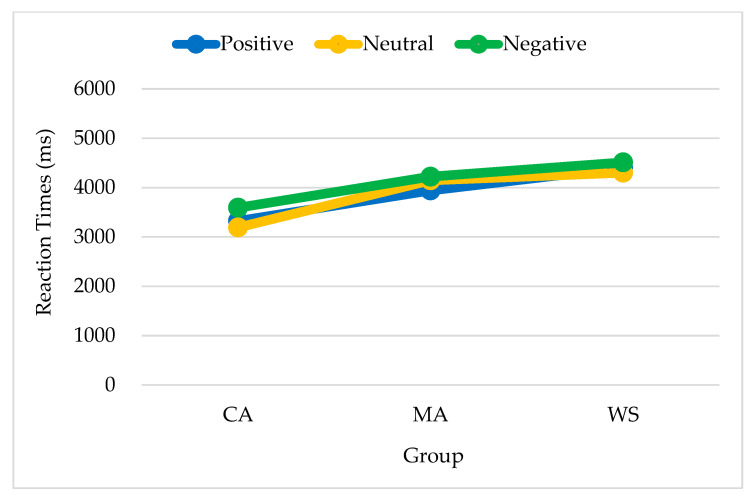
Reaction times of the responses to negative primes by emotion target type and group. Note. CA, chronological age-matched controls; MA, mental age-matched controls; WS, participants with Williams syndrome.

In summary, regarding responses to positive and neutral emotions, the people with WS responded similarly to the CA controls and differed in both from the responses of the MA controls. The reaction time patterns for positive, neutral, and negative primes are shown in Figure 4, Figure 5 and Figure 6 respectively.

## 4. Discussion

This study investigated how people with WS categorize emotions via priming. By presenting three types of emotion primes (positive, neutral, and negative), responses to the same three emotion targets were measured and analyzed. People with WS showed patterns of emotion priming similar to those of typically developing controls but with lower accuracy. Under positive-prime conditions, both positive and negative emotion targets were primed more accurately than neutral targets. Under neutral- and negative-prime conditions, people with WS confused positive and neutral emotion targets because they potentially perceived these two targets as being similar. However, compared with the accuracy for the two other emotion target types, the accuracy for negative emotion targets was significantly higher and significantly lower in the neutral- and negative-prime conditions, respectively. In all prime conditions, people with WS generally responded less accurately than the matched controls, although these differences only reached significance in the positive-prime conditions. Taken together, people with WS categorized positive and negative emotions and matched typically developing controls, albeit with greater confusion when categorizing neutral and positive emotions.

This study contributes to advancing our understanding of the emotional knowledge of people with WS. Considering the hierarchy of emotions, this study uncovered the classification of subordinate levels of emotion in people with WS. Hsu and Lv [11] examined the ability of people with WS to recognize basic emotions and showed their delayed performance in labeling *angry* and *surprised* emotions. Through this follow-up study on basic emotions, we have expanded our knowledge about the hierarchy of basic emotions in people with WS.

In research by Rose et al. [21], the findings showed that people with WS tended to use a feature-based strategy to process faces with and without emotions. With this strategy, people with WS showed a strong inversion effect (i.e., using a feature-based strategy to process faces rather than using a configuration-based strategy on inverted faces); however, they were less accurate in processing faces with emotions that were changed in configurations. In Rose et al. [21], people with WS, people with autism, and typically developing controls were tested on their judgments of three types of faces (upright neutral, upright affect, and inverted neutral), and their face processing abilities were assessed. For upright, neutral faces, all three groups showed high accuracy; people with WS had a stronger inversion effect than typically developing controls, and people with autism showed the strongest inversion effect among the groups. Further, for upright affected faces, people with WS showed higher accuracy than for inverted neutral faces, whereas people with autism showed the lowest accuracy in identifying upright affected faces. These findings indicate that people with WS were atypical in integrating contextual information. In turn, this atypical contextual information integration may have influenced the categorization of emotions by people with WS.

When Jarrold et al. [22] investigated the conceptual structure of people with WS by examining their semantic fluency through the production of categories (i.e., animals and body parts) in a limited time, the authors observed that their conceptual structure was atypical. Specifically, the authors tested groups of people with WS, people with learning difficulties, and typically developing controls. Their results revealed that people with WS generally did not differ from other groups in the frequency, typicality, and number of productions in category-related item production. However, the order of item production differed in people with WS, showing more repetitions than in typically developing controls. Jarrold et al. [22] also analyzed the distances among category items, using them as indices to represent the semantic closeness among items. People with WS seemed to be less sophisticated in their conceptual structures than people with learning difficulties and the typically developing controls. The current findings coincide with those of Jarrold et al. [22] in that the gross semantic structure related to emotions in the valence categorization in people with WS was not atypical; however, their response latency was much slower, and their accuracy was lower than that of the typically developing controls.

People with WS have also been reported to be atypical in perceiving faces judged as having low approachability [23]. Bellugi et al. [23] asked participants to judge whether the faces displayed were approachable on a scale from −2 (negative feelings toward the face) to +2 (positive feelings toward the face) and revealed that people with WS responded to faces that were negatively judged as having low approachability with high scores. This finding suggests that people with WS have different sensitivities to faces than typically-developing controls. Another study by Fidler et al. [24] confirmed the enhanced intersubjectivity and emotional responsiveness of people with WS compared to a group with mixed etiologies (e.g., Down syndrome, Angelman’s syndrome), including negative and positive emotions in facial display and vocal production. This atypical processing of faces in individuals with WS could result from deficiencies in contextual integration, which have been shown in the verbal studies by Hsu et al. [25], Hsu and Tzeng [26], and Hsu [20,27], as well as the nonverbal studies by Hsu [28,29,30], and Hsu and Chen [31].

In the verbal studies, people with WS showed atypical behaviors when integrating propositions [26], forming themes from related associates [25], and priming words with functional relatedness [20]. Further, people with WS showed delays in their causal inferences [27] and when priming words with synonymous and categorical relations. In the nonverbal studies, people with WS showed delayed performances in contextual integration via background pictures and target objects in their visual [29], auditory [30], and cross-modal [28] forms. Two other studies show that their neural processing of semantically related words and face processing were also atypical [25,31]. In this study, different emotion types were used as contextual primes and were coupled with different emotion types as contextual targets. Consequently, people with WS gave significantly less accurate responses than the typically developing controls. This result again supports the weak contextual integration of this population.

An interesting finding of this study is the negative priming effect that appeared when people with WS judged negative primes with negative emotion targets. This finding might be related to the working memory capacity of people with WS when processing mixed negative emotions. In Megias et al. [32], the negative priming effect was observed for semantically unrelated words with semantically related words when delay masking was used. However, no negative priming effect was observed when the immediate masking method was used. Moreover, only people with high (vs. low) working memory capacity showed negative priming. Megias et al. [32] explained the negative priming effect as a result of both the excitatory effect of semantically related targets and the inhibitory effect of the ignored primes, regardless of their semantic relatedness. Considering that this study used emotion category priming (not word-pair semantic priming), it may be that the negative priming effect arose from the concomitant inhibition of the emotion primes and the excitation of the emotion targets.

This negative priming effect only appeared for negative emotions in this study. This effect could be explained by the recency effect in memory [33], specifically, when previously learned items influence newly learned information. This influence may have caused the negative priming effect to be the largest among people with WS when testing emotion category priming. Another possible explanation for this effect may be related to the mixing of emotions of the same valence (e.g., negative emotions such as *anger*, *sadness*, *fear*, and *disgust*), as distinct emotions of negative valence might have hindered the emotion categorization of all three analyzed groups.

This study aimed to determine whether the emotion categorization of people with WS was typical, delayed, or atypical compared with typically developing controls. In the emotion priming task, although people with WS showed significantly lower accuracy, they could classify emotions (i.e., positive, neutral, and negative) similar to the typically developing controls. This finding of lower accuracy in people with WS confirmed the proposal of pure lexical enrichment with weak conceptual change in this population [34], which is hypothesized as the root of superficial understanding in the language production and comprehension of people with WS. Conceptual change requires the process of differentiating words in lexical semantics, such as inductive inferences. People with WS lack the process of differentiation; hence, a deep understanding of lexical semantics is deficient in this group. This study contributes to the knowledge of emotion categorization in people with WS through the understanding of affective words. Based on Fisher’s model of emotion hierarchy, people with WS showed mixed emotion recognition from a superordinate perspective [8] and deviant replacement patterns for the basic emotions [11]. This study demonstrated that people with WS were generally less accurate in categorizing emotions from the subordinate perspective of lexical words. The rationale is clear, and the hypothesis was confirmed—that people with WS have superficial semantic knowledge. This is probably one of the root causes of the deficient contextual integration abilities of people with WS.

This study not only advances our knowledge of the emotion categorization of people with WS but also confirms the hierarchical emotion model [2]. This study also lends support to the understanding that people with WS have, to a certain extent, an atypical perception of social emotions. Future studies may examine whether the generally similar pattern in emotion priming revealed by people with WS as compared to developed controls could be observed at the neurological level. Based on the brain and behavioral asymmetry hypothesis, it is predicted that deviant brainwaves in responses to subordinate types of emotions would be displayed in people with WS. Furthermore, some emotional words belong to one of the basic emotions; others belong to complex emotions (i.e., combinations of the basic emotions). More finely-controlled stimuli across subcategories in each emotional valence could be considered.

## Figures and Tables

**Table 1:** Background information on participants.

Group	N (F:M)	CA	CA Range (SD)	MA	MA Range (SD)
CA	14 (3:11)	10.24	7.03–17.07 (2.61)	---	---
MA	14 (3:11)	6.44	4.02–11.09 (2.09)	---	---
WS	14 (3:11)	10.49	7.02–17.01 (2.44)	6.57	4.05–11.06 (1.89)
College rating	20 (10:10)	20.88	17.11–24.01 (2.26)	---	---
College ISI 250 ms	20 (10:10)	23.56	19.10–26.01 (1.80)	---	---
College ISI 750 ms	20 (10:10)	22.81	19.07–26.01 (2.30)	---	---

Note. CA, chronological age-matched controls; MA, mental age-matched controls; WS, participants with Williams syndrome; ISI, inter-stimulus interval; SD, standard deviation; F, female; and M, male.

**Table 2:** Matrix of accuracy for the three groups in responses to three types of targets given the three types of primes.

Accuracy for positive primes (SE) [*F*_(2,38)_ = 11.704, *p* < 0.001, *η*² = 0.381]			
Target types	CA	MA	WS
Positives	0.875 (0.050)	0.800 (0.052)	0.711 (0.050)
Neutrals	0.771 (0.067)	0.704 (0.069)	0.464 (0.067)
Negatives	0.871 (0.050)	0.854 (0.052)	0.700 (0.050)
Accuracy for neutral primes (SE) [*F* < 1]			
Positives	0.668 (0.071)	0.614 (0.071)	0.385 (0.084)
Neutrals	0.693 (0.068)	0.686 (0.068)	0.675 (0.080)
Negatives	0.800 (0.047)	0.804 (0.047)	0.800 (0.056)
Accuracy for negative primes (SE) [*F*_(2,38)_ = 3.208, *p* = 0.052, *η*² = 0.144]			
Positives	0.793 (0.064)	0.704 (0.064)	0.573 (0.066)
Neutrals	0.779 (0.061)	0.718 (0.061)	0.573 (0.063)
Negatives	0.525 (0.076)	0.429 (0.076)	0.462 (0.078)

Note. CA, chronological age-matched controls; MA, mental age-matched controls; WS, participants with Williams syndrome.

**Table 3:** Matrix of reaction times for the three groups in responses to three types of targets given the three types of primes.

Reaction times for positive primes (SE) [*F*_(2,38)_ = 6.267, *p* = 0.004, *η²* = 0.248]			
Target types	CA	MA	WS
Positives	2753.455 (520.021)	4759.749 (539.651)	3352.731 (520.021)
Neutrals	2955.582 (470.654)	4971.511 (488.421)	3709.164 (470.654)
Negatives	2970.044 (483.377)	5480.306 (501.624)	3163.570 (483.377)
Reaction times for neutral primes (SE) [*F*_(2,35)_ = 5.614, *p* = 0.008, *η*² = 0.243]			
Positives	3230.414 (500.281)	5035.356 (500.281)	3854.254 (591.941)
Neutrals	3137.287 (395.650)	5044.330 (395.650)	3482.550 (468.139)
Negatives	3190.631 (427.821)	5032.522 (427.821)	3372.084 (506.204)
Reaction times for negative primes (SE) [*F* < 1]			
Positives	3323.979 (554.670)	3941.400 (554.670)	4402.384 (554.670)
Neutrals	3193.843 (544.686)	4147.069 (544.686)	4300.490 (544.686)
Negatives	3593.080 (608.957)	4220.744 (608.957)	4512.716 (608.957)

Note. CA, chronological age-matched controls; MA, mental age-matched controls; WS, participants with Williams syndrome.

## Data Availability

Data are available on request.
